# Supplementary material for: Active site specificity profiling datasets of matrix metalloproteinases (MMPs) 1, 2, 3, 7, 8, 9, 12, 13 and 14
Source: Data Brief. 2016 Feb 22;7:299–310. doi: 10.1016/j.dib.2016.02.036 (PMC4777984; doi:10.1016/j.dib.2016.02.036)
Supplement: Supplementary file 10 — Supplementary material [file mmc10.zip › WebPICS_hMMP7_T_1%/P1.html]

 

PICS results


|  |  |
| --- | --- |
| **P1\_D**  26 in 291 sites   8.9 %    effects > 10 perc. pnts.  (vice-versa in brackets)  P2\_E: 15.9 (13.0) |  |
| **P1\_E**  37 in 291 sites   12.7 %    effects > 10 perc. pnts.  (vice-versa in brackets)  P2\_A: 11.6 (11.6)   P3prime\_Q: 14.7 (27.3) |  |
| **P1\_F**  16 in 291 sites   5.5 %    effects > 10 perc. pnts.  (vice-versa in brackets)  P2\_D: 16.4 (10.5)   P3prime\_H: 14.7 (19.5) |  |
| **P1\_G**  25 in 291 sites   8.6 %    effects > 10 perc. pnts.  (vice-versa in brackets)  P2\_G: 25.0 (19.5) |  |
| **P1\_H**  10 in 291 sites   3.4 %    effects > 10 perc. pnts.  (vice-versa in brackets)  P3prime\_N: 15.5 (12.0) |  |
| **P1\_N**  33 in 291 sites   11.3 %    effects > 10 perc. pnts.  (vice-versa in brackets)  P2\_Y: 11.8 (38.7) |  |
| **P1\_P**  25 in 291 sites   8.6 %    effects > 10 perc. pnts.  (vice-versa in brackets)  P3prime\_D: 12.4 (14.1) |  |
| **P1\_Q**  22 in 291 sites   7.6 %    effects > 10 perc. pnts.  (vice-versa in brackets)  P2prime\_T: 12.0 (14.6) |  |
| **P1\_S**  24 in 291 sites   8.2 %    effects > 10 perc. pnts.  (vice-versa in brackets)  P3\_A: 17.2 (11.8)   P1prime\_M: 12.2 (22.6)   P3prime\_G: 16.8 (11.2) |  |
